# Enhancing Perception with Tactile Object Recognition in Adaptive Grippers for Human–Robot Interaction

**DOI:** 10.3390/s18030692

**Published:** 2018-02-26

**Authors:** Juan M. Gandarias, Jesús M. Gómez-de-Gabriel, Alfonso J. García-Cerezo

**Affiliations:** System Engineering and Automation Department, University of Málaga, 29071 Málaga, Spain; jmgandarias@uma.es (J.M.G.); ajgarcia@uma.es (A.J.G.-C.)

**Keywords:** adaptive gripper, tactile sensors, human–robot interaction, object recognition

## Abstract

The use of tactile perception can help first response robotic teams in disaster scenarios, where visibility conditions are often reduced due to the presence of dust, mud, or smoke, distinguishing human limbs from other objects with similar shapes. Here, the integration of the tactile sensor in adaptive grippers is evaluated, measuring the performance of an object recognition task based on deep convolutional neural networks (DCNNs) using a flexible sensor mounted in adaptive grippers. A total of 15 classes with 50 tactile images each were trained, including human body parts and common environment objects, in semi-rigid and flexible adaptive grippers based on the fin ray effect. The classifier was compared against the rigid configuration and a support vector machine classifier (SVM). Finally, a two-level output network has been proposed to provide both object-type recognition and human/non-human classification. Sensors in adaptive grippers have a higher number of non-null tactels (up to 37% more), with a lower mean of pressure values (up to 72% less) than when using a rigid sensor, with a softer grip, which is needed in physical human–robot interaction (pHRI). A semi-rigid implementation with 95.13% object recognition rate was chosen, even though the human/non-human classification had better results (98.78%) with a rigid sensor.

## 1. Introduction

Haptic perception, conceived as a combination of kinesthetic and tactile information, is indispensable for human beings. Daily living activities such as manipulating and grasping objects would be impossible without the sense of touch [1]. As in humans, tactile information is useful in robotics in different ways, such as for object recognition or grasping tasks, physical human–robot interaction (pHRI), or dexterous manipulation [2,3].

It is becoming more common to see the use of robots in different applications. In those in which the interaction of the robot with the environment is required, tactile perception is critical. In robotics surgery, tactile sensing plays an important role [4,5,6]. Moreover, in other applications related to medicine (e.g., for rehabilitation [7,8] or biomedical engineering [9]), tactile sensing is often used. The use of tactile perception in pHRI operations for touching or manipulating humans has also been considered [10]. In [11], a textile pressure sensor was developed to detect the emotional intention of human touches.

**Figure 1:**
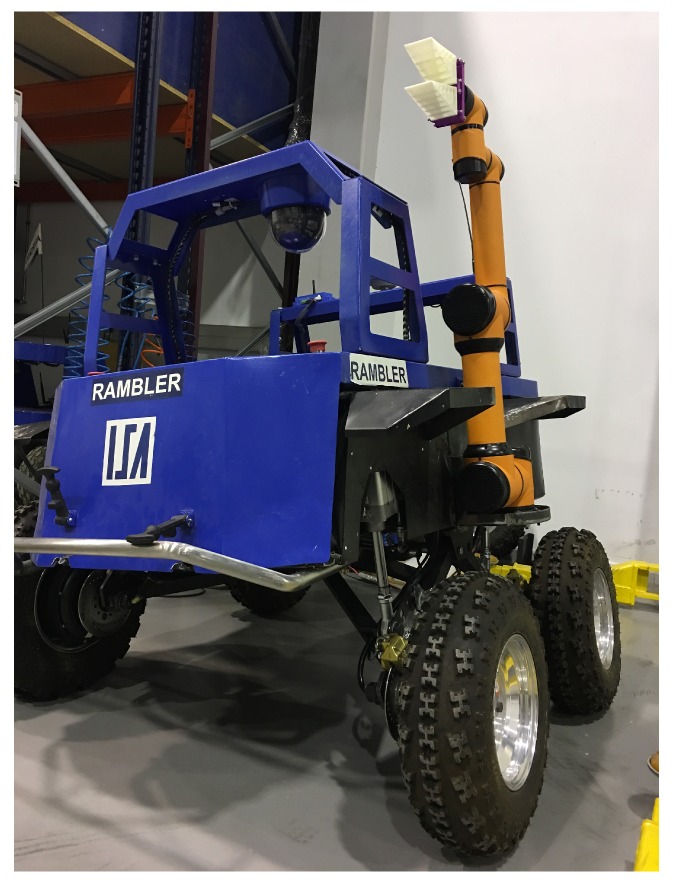
The all-terrain mobile robot for rescue applications, from the University of Málaga, showing the attached arm with a flexible adaptive gripper.

Autonomous or teleoperated first response robotics (as the robot show in Figure 1) for emergency situations operate in disaster scenarios where visibility conditions can be reduced due to the presence of dust, mud, or smoke, revealing the limitations of existing emergency robots. A mobile manipulator should be able to identify objects with similar shapes. With this ability, a robot with tactile perception can enhance the perception capabilities of the system, whether teleoperated or autonomous. Moreover, the use of a suitable gripper is important to avoid injuring possible humans. Identification of the manipulated object using soft or adaptive grippers is important for a safe physical human–robot interaction (pHRI).

Tactile identification of the objects in uncertain environments is complex due to the variability of the objects and grasping conditions. In particular, the size of the detected contact area—even for the same object type—can change depending on multiple factors:

- Location of the object inside the gripper. As the sensing area is often smaller than the contact surface, the detected contact area can be different. A diagonal alignment with the sensor provides larger area than a vertical (or horizontal) one.
- Stiffness of the object and the gripper. Soft objects change the contact area depending on the pressure. Rigid objects and rigid grippers provide higher pressures with smaller contact areas. The contact pressure depends on the gripping force, but also on the object weight and eventual external forces.
- Size of the object. Individuals of the same object class may have different sizes. Human body parts can also be found in a variety of sizes depending on the sex, age, and physical condition [12,13] of the subject, providing similar contact pressure shapes with different scales.

As the size of the contact area cannot be used to reliably classify the grasped object, the use of deep convolutional neural networks (DCNNs) designed and trained for computer vision applications may provide accurate object recognition using tactile images with independence of the location, scale, and overall pressure.

In this work, two types of adaptive grippers for pHRI were built, and a flexible high-resolution tactile sensor was integrated to measure the performance of the manipulator in the classification of the grasped objects, based on DCNN. The set of training objects included human limbs and common objects, so a dual classification was performed based on the type of object and on its human nature. The results of the different approaches are compared to identify the best configuration for the desired task.

To our best knowledge, no previous work has integrated a flexible tactile sensor within adaptive grippers for object recognition. The major contribution and the novelty of this work lies in the first evaluation of different gripper hardness for tactile recognition with a flexible tactile sensor. To this end, we first analysed the sensing characteristics of two types of adaptive grippers and their suitability for pHRI applications. Next, we propose the adoption of a method based on a pre-trained deep convolutional neural network to classify different types of objects and human body parts. Furthermore, the paper offers experimental results indicating that the proposed solution can be applied to disaster response robots to better support rescue workers in future emergencies.

In the next section, a review of the related works is included. In Section 3, the sensor and the configuration of the grippers is described. Section 4 describes the implemented classification methods. In Section 5, the comparative experiments are included. Section 6 offers a discussion based on the experimental results, and the paper is closed with a Conclusions section.

## 2. Related Work

Different topics related to the use of tactile sensors in robot grippers and tactile object recognition for pHRI applications have been reviewed.

### 2.1. Human–Robot Interaction

In the case of interaction with humans, the robot is required to manipulate or grasp parts of the human body (e.g., hands, arms, head). In those situations where pHRI is required, the safety is indispensable [14]. In [15], an overview of the use of capacitive tactile proximity sensors (CTPSs) for different applications (i.e., grasping, haptic exploration, collision avoidance, and safe human–robot interaction) is presented. We need to exert low pressure, so large fingers are better suited for this task.

Flexible fingers can adapt better to the shape of the body part to provide a soft contact but limit the maximum gripping forces, as shown in [16]. In this paper, soft robotics grippers are used to delicately manipulate fragile species on deep reefs.

The use of adaptive or soft robots has also been applied for medical activities. In [17], a soft robotic glove for rehabilitation utilizes soft actuators that induce specific bending trajectories that are able to produce significant forces under controlled pressures and present low impedances when un-actuated to allow the movement of the fingers without risk of injuring the patient.

### 2.2. Haptic Perception in Adaptive and Soft Grippers

Recent trends in robotics are based on the use of soft or adaptive grippers [18,19]. Related works present the integration of sensors with haptic feedback. Although soft grippers have a simple structure, good compliance, and a natural motion, there are still significant challenges about the sensors used for detecting forces and deformations. Those points are considered in [20], where a pneumatic soft sensor is used for measuring the contact force and curvature of a soft gripper.

Soft robotics grippers are used to identify objects through haptic perception. In [21], a self-adaptive under-actuated gripper with haptic feedback is presented. On the other hand, a soft gripper is employed to identify objects in [22]. Each finger of the three-finger gripper has resistive bend sensors to provide a configuration estimate for distinguishing the grasped object using a clustering algorithm.

### 2.3. Robot Grasping

The use of a tactile sensor inside the gripper provides useful information for gasping [23,24], object recognition [25], and manipulation [26,27] tasks. This problem has not yet been solved [28].

Multiple research projects have based their perception of the robots on the information provided by force or tactile sensors for carrying out grasping and manipulation tasks [29]. One common approach in this research line consists of estimating the pose of the grasped object based upon tactile information. In [30], the design of a tactile sensor to estimate the pose and orientations of a grasped wire is presented. Other approaches presented in [31] are based on exploiting the contact and position information to estimate the pose of an object inside a robotic hand, then a bootstrap particle filter is used to evaluate multiple hypotheses of the estimation of the pose.

The use of simple rigid sensors provides little contact information, so many articulated rigid grippers have multi-pad tactile sensors to perceive different areas of the grasped object [32].

### 2.4. Tactile Object Recognition

In the literature, multiple research projects related to object recognition can be found. However, given the differences in the hardware, providing a comparison between the different works is complex [32]. Essentially, two main approaches are defined. On one hand, object recognition is carried out by identifying the material or the surface of the object in contact with the robot [33,34]. On the other hand, the recognition is based on the shape of the object [25,35,36,37].

Independent of the approach, the majority of these works are based on the use of artificial intelligence algorithms to classify the data. One proposition consists of using computer vision algorithms and machine learning techniques by treating the pressure data as images [38,39]. Moreover, related works made use of neural networks and deep learning [40,41]. One of the benefits of using convolutional-networks-based techniques is the translation-invariant property presented in this kind of neural network [42]. In [43], a DCNN-based algorithm to recognise an in-contact human hand with a robotic arm covered by an artificial skin is developed. This algorithm takes advantage of the translation-invariant property of the network, and is able to recognise both left and right hands with different postures.

Other object recognition concepts can be found. In [44], the benefits of using both kinesthetic and tactile information is presented. On the other hand, in [45,46] the interpretation of the tactile data as time series is considered. The use of sequential data is important to further identify properties of the objects (e.g., textures). In the case of [47], an exploratory motion is performed to obtain dynamic tactile information with a single force sensor (pressure and tangential compliance). In that case, the control of the actuator is important to keep constant pressure against the materials and obtain reliable information. The classifier uses a multi-channel approach with very high accuracy. In [48], a flexible high-resolution sensor was used to create test equipment to evaluate food textures. They use a DCNN with a pressure sequence image, taken as the artificial mouth closes, to identify parameters of the food’s texture. The sequence of tactile images is important to track the behaviour of gel-like food when breaking, even though they found that using only two pressure images yields similar results.

Multi-modal techniques were considered in [49]. Approaches based on the fusion of haptic and visual data provide better results than using a single type of information, and make a robot able to predict the physical interaction with the object just by “seeing” it [50].

## 3. Sensor and Gripper Configurations

This section includes a description of the tactile sensor used and the characteristics of the two adaptive grippers, with a comparative study of the sensing differences between using a rigid sensor probe and the two grippers.

### 3.1. Tactile Sensor

The data acquisition system is composed of the tactile sensor, model 5051 from *Tekscan* (South Boston, MA, USA). The high-resolution tactile-array has a total of 1936 pressure sensels (also called tactels or taxels), where each one has a size of 0.76×0.76 mm and the sensor presents a density of $62\;\mathrm{tactels}/{\mathrm{cm}}^{2}$ distributed in a matrix of 44 rows by 44 columns of resistive pressure sensors. In Table 1, the main features of the sensor are shown. Data acquisition software provided by *Tekscan* was used.

A silicon rubber pad (manufactured by *RS* with code 733-6713) provides soft contact between the sensor and the objects. Its main mechanical properties are summarized in Table 2.

### 3.2. Adaptive Grippers

**Figure 2:**
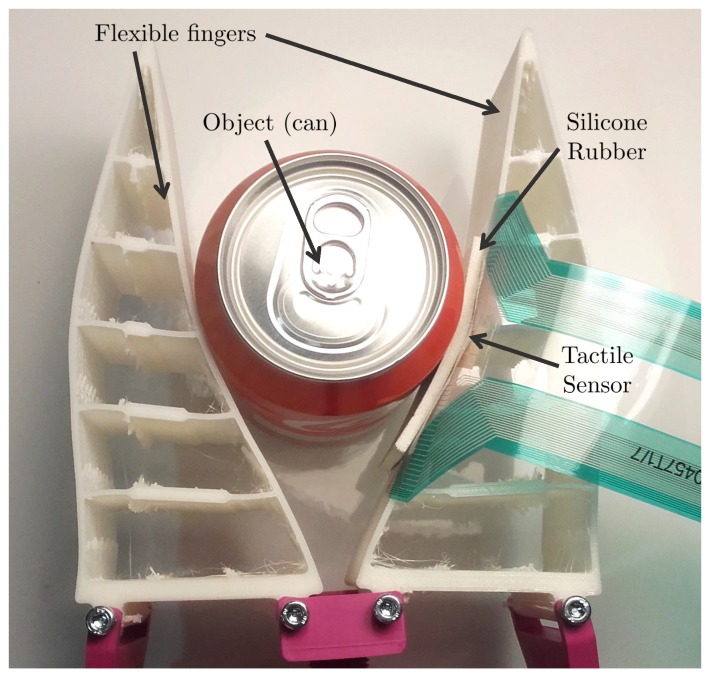
Adaptive gripper based on the Fin Ray^®^ effect with the flexible configuration and a tactile sensor. Note that the tactile sensor bends to the contact surface due to the structure of the fingers, changing the perceived information.

Two adaptive grippers were implemented based on a two-finger version of the adaptive griper manufactured by *Festo* (https://www.festo.com/group/en/cms/10221.htm) using the *Fin Ray*^®^ effect, as shown in Figure 2. This kind of mechanism can be built with different materials, and exhibits an interesting bending behaviour [51]. The tactile sensor pad is located on the inner side of one of the fingers, and bends as the finger adapts to the contact surface.

**Figure 3:**
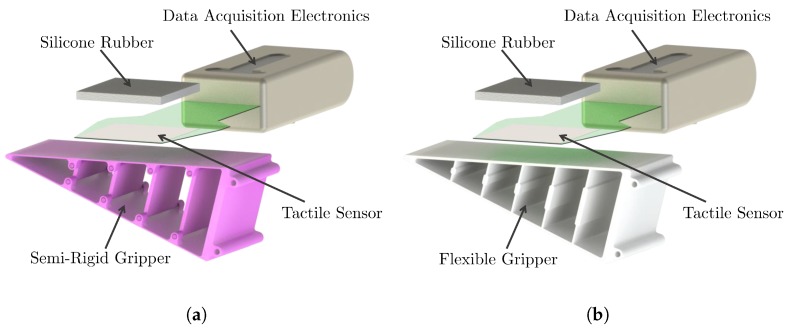
Illustration of (**a**) Semi-rigid and (**b**) flexible finger and sensor mounting configurations, showing the dispositions of the data acquisition electronics and the contact rubber pad. Note that the structure of the semi-rigid gripper has joints that allow the bending of the surface.

Different materials were used with similar designs (https://www.thingiverse.com/thing:2710725) using Fused Deposition Modeling (FDM) 3D printing. The materials used are PLA (PolyLactic Acid) for the semi-rigid gripper, and thermoplastic elastomer (poliurethane) with hardness 90A for the flexible gripper, by *Smart Materials 3D (Spain)*. Figure 3 shows the rendered image of the CAD models for each configuration, including the attached tactile sensor.

The semi-rigid gripper has a multi-part design with hinged ribs than can be 3D-printed in plastic and assembled in a single batch. The sides of the fingers have a thickness of 0.8 mm to make it bendable. The flexible version is designed as a single part using elastomer material. The ribs have a thicker middle section to provide the desired bending effect.

### 3.3. Mechanical Properties

**Figure 4:**
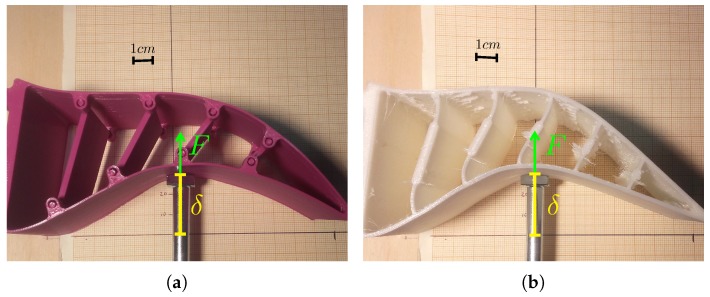
Experimental measurement of the fingers’ elasticity (without tactile sensor and pad) through the displacements δ produced by a controlled force *F* with (**a**) the semi-rigid configuration and (**b**) with the flexible configuration.

To characterize soft materials or grippers, the force/displacement relationship was measured. Figure 4 shows the characterization process of the adaptive fingers’ elasticity before mounting the tactile sensor. In this process, a force sensor with a range of 0.01 to 50 zN, similar to the force sensor used in [52], was used.

**Figure 5:**
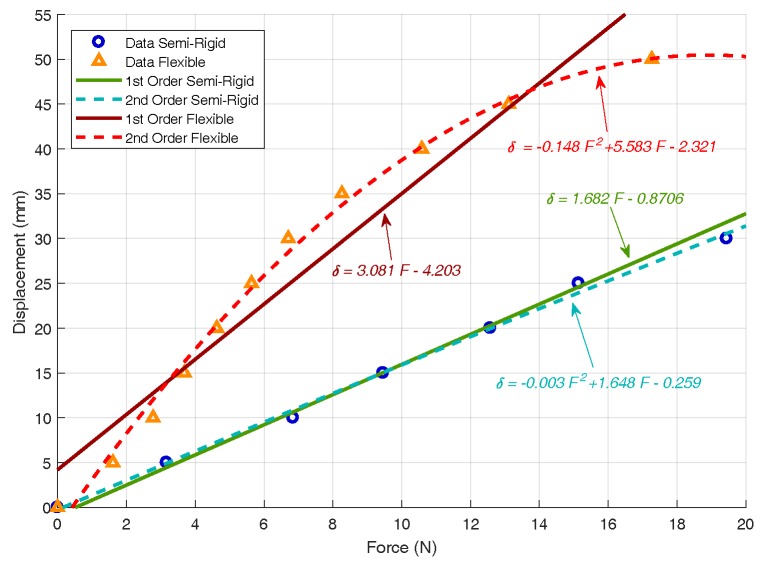
Parameter identification of the linear and quadratic models of the semi-rigid and flexible configurations.

Figure 5 shows the force/displacement curves and the identified parameters, based on a first- and second-order polynomial regression using the *Curve Fitting Toolbox* from *Matlab*. Note that the models are valid for the range of measured forces of up to 20 N, which seems to be the maximum force before damaging the finger or losing its bending behaviour.

### 3.4. Different Tactile Images

**Figure 6:**
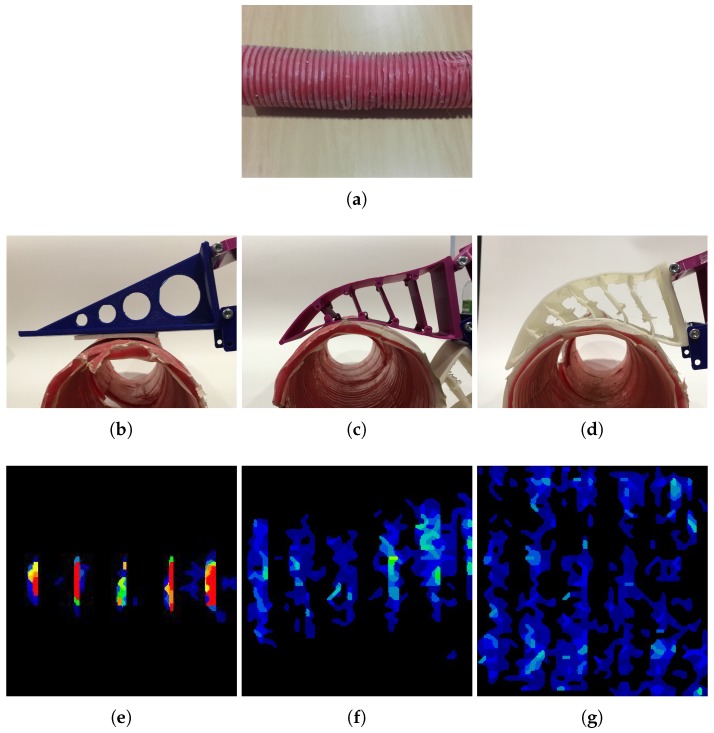
Different contact areas of the different gripper configurations, showing the fin ray effect. (**a**) Plastic pipe object, (**b**) Rigid contact, (**c**) Semi-rigid gripper contact, (**d**) Flexible gripper contact, (**e**) Pressure image from contact with the rigid probe, (**f**) Pressure image from contact with the semi-rigid gripper and (**g**) Pressure image from contact with the flexible gripper.

An adaptive gripper increases the contact surface between the object and the gripper. When the contact surface is larger, the information acquired by the tactile sensor is higher. To illustrate the differences, a sample object was used. In Figure 6, a picture of a plastic pipe is shown, along with the corresponding pressure images obtained with each configuration.

The contact surface can be calculated using Equation (1), where *S* is the contact surface, κ is the density of the sensor, measured as the number of tactels per square centimeter, and *n* is the number of tactels with values other than 0.

(1) $$S=\frac{n}{\kappa }$$

The mean value perceived by the tactels in contact with an object can be obtained according to Equation (2), where $\mu_{p}$ is the mean value of the tactels in contact, and p(i,j) is the value of the tactel (i,j).

(2) $$\mu_{p}=\frac{\sum_{i,j=1,1}^{m,n}p(i,j)}{n}$$

**Figure 7:**
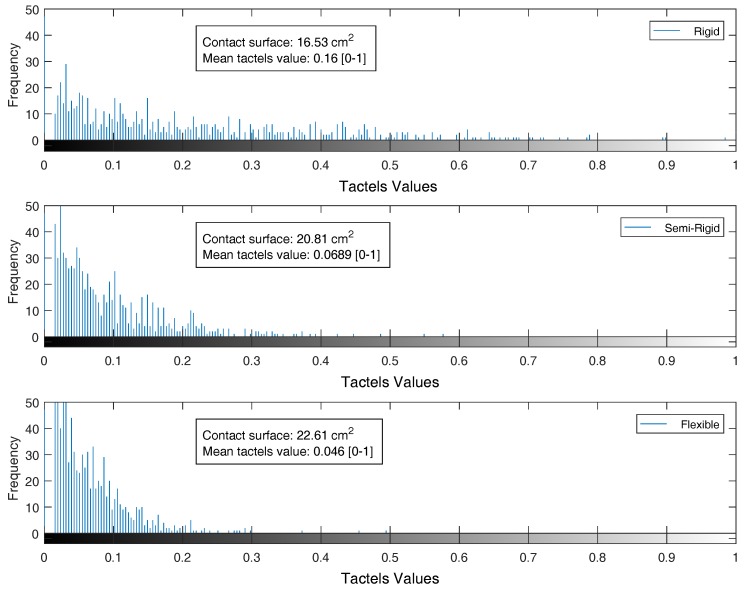
Histograms with contact surface and mean tactels value of the data recorded while touching the object in Figure 6 with each configuration.

The histograms of the tactile images exhibit information about the distribution of the pressure values. For the sample tactile images presented in Figure 6, the histogram and the mean value of the tactels is shown in Figure 7. The contact area in the sensor of a semi-rigid gripper was 26% greater than the contact area in the rigid sensor, and 37% in the case of the flexible gripper. The mean pressure value at the semi-rigid sensor was 57% lower than the mean pressure at the rigid probe, and 72% lower in the case of the flexible gripper. Even when the tactile sensor did not cover all of the finger surface, the detected contact area of the rigid probe was smaller than in the adaptive grippers. The flexible gripper measured the largest contact surface when touching the same object. Nonetheless, the histograms related to adaptive grippers present a lower range of pressure values than the rigid probe.

## 4. Tactile Recognition

The recognition procedure adopted in this paper complies with the idea of classifying an object by its contact shape, treating pressure data from a high-resolution tactile sensor as common images, and using a deep convolutional neural network [53]. This method was successfully implemented with a tactile sensor mounted on a rigid probe, applied to robotics search and rescue tasks [54], showing how tactile perception provides valuable information for the search of potential victims—especially in low-visibility scenarios. The purpose of this classifier is to augment the sensory perception by providing cognitive functions to a robotic system that can be teleoperated in emergency situations where the visibility conditions are poor due to the presence of dust, mud, or smoke, and the best way to identify the nature of objects with similar shapes is by using its tactile properties.

The tactile object recognition is implemented in two stages. First, the convolutional layers of a pre-trained DCNN extract the features from the input tactile data. Next, custom-made layers are introduced at the final-stage of the network to classify according to our predefined class in a supervised learning process. Moreover, a variation of this methodology which consists of a DCNN with two levels of classification is implemented. This way, the first-level classifier provides the nature of the object in contact (in our case, a human limb or a common object), whereas the second level provides the object label of the contact.

### 4.1. Dataset

**Figure 8:**
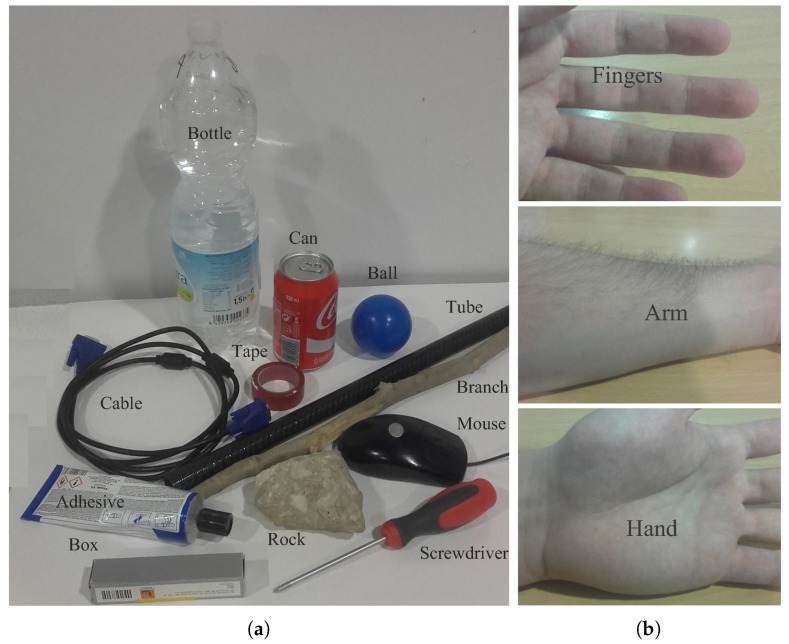
Images of (**a**) the inert objects and (**b**) human limbs used for the experiments.

For the classification process, a dataset of labelled data is necessary. The dataset is composed of normalized pressure images from 15 classes of objects, including 12 from ordinary objects and three from human body parts. A picture of the objects and the body parts used in the experiments is shown in Figure 8. As the recognition can be done on two levels, the objects were labelled in two ways: first by their human nature, and second as individual objects, as shown in Table 3.

**Figure 9:**
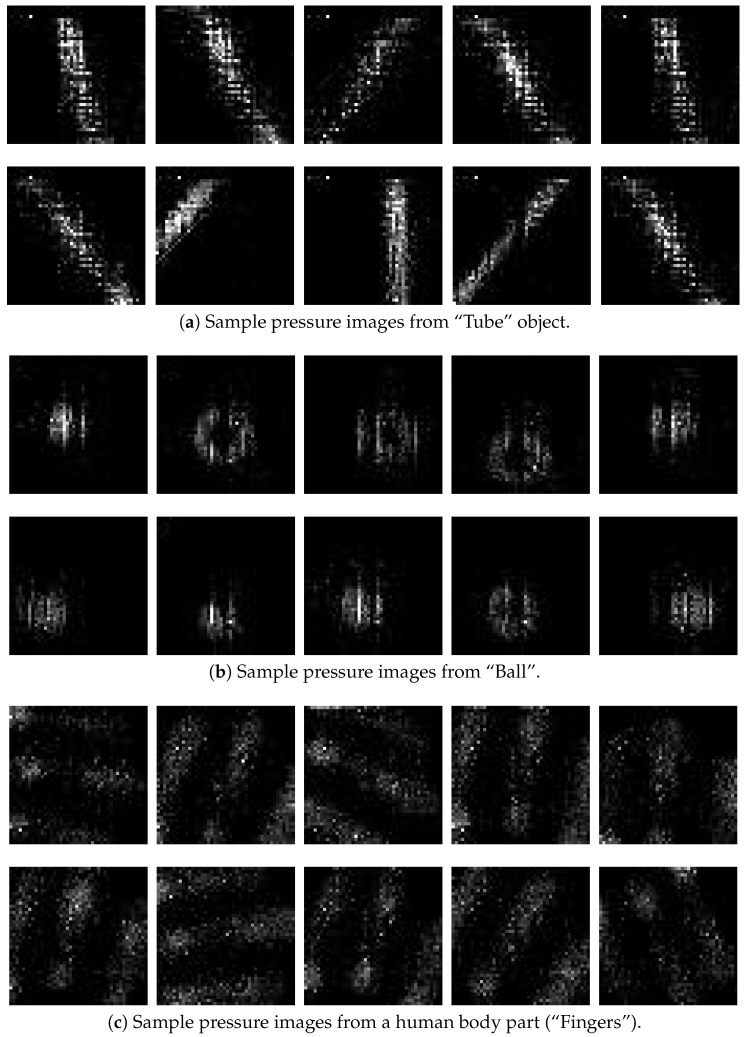
Sample of pressure images from the dataset created for the experiments taken with the flexible adaptive gripper. (**a**) two rows from the class *tube*, (**b**) the two middle rows are from the class *ball*, and (**c**) the two last rows are from the class *fingers*. Each training class has a total of 50 pressure images for each type of gripper for the objects detailed in Table 3.

The reference tactile images were recorded by manually grasping the objects with the three gripper configurations, as shown in Figure 6b–d. For each configuration, 50 tactile images for each class were obtained, giving a total of 750 images each. In Figure 9, a sample of 30 pressure images of the classes *tube*, *ball*, and *fingers* (10 for each class) are shown. The images were collected using the flexible gripper.

**Figure 10:**
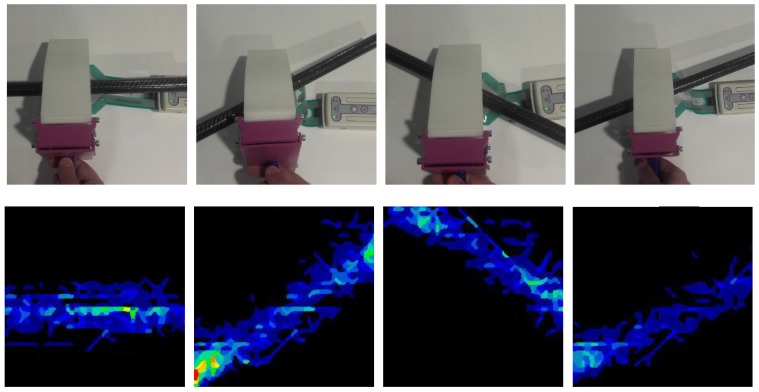
Illustration of the data collection procedure by grasping an object (tube) from different locations with the flexible gripper (**top**) and the corresponding pressure images (**bottom**).

As the convolutional-network-based methods used for the experiments are translation-invariant, the data capture both for the training and validation sets was carried out by grasping the objects with different positions and orientations. The *I-Scan* software from *Tekscan* was used to start recording a sequence of measurements. Then, the gripper was used to repeatedly close and open up to 200 times over the target object, which was manually placed by the operator in random positions trying to keep the centre of the object grasping area over the sensing surface (55.9×55.9 mm). While placing the object, the operator also changed the pitch angle randomly with an angle from −45 to 45 degrees between the object’s main axis and the bending axis of the gripper. There was no roll angle variation. Then, the recording was stopped and the sequence was reviewed with the same software and 50 tactile images were manually chosen and saved as individual *.csv* files. After that, the files were converted to *.jpg* files using a *matlab* script. Finally, the full set of images was directly used to feed the classification methods using the *Neural Network Toolbox*. An illustration of the data collection procedure is presented in Figure 10. The fin ray effect gripper pushes the objects to the middle section of the gripper. However, if the object is fixed, a compliant position control of the gripper is assumed, with the help of force/torque sensors.

### 4.2. Tactile Data Classification with a DCNN

The DCNN used in this work is based on *AlexNet*, a highly used neural network in computer vision for object classification [55]. This network contains eight layers, where the first five are convolutional and the last three are fully-connected.

A large dataset is needed to train this kind of neural network. Although there is a wide variety of datasets with common RGB images, there is no dataset of tactile images which fit in our application. Creating a large enough dataset with labelled tactile data to train this kind of DCNN is an arduous task. Instead, we propose the use of the transfer learning approach. This concept is based on the idea of pre-training the DCNN in a dataset of RGB images and then only re-training the final layers, so a smaller tactile dataset is needed. The network was pre-trained in the *Imagenet* dataset [56], formed by common pictures (RGB). Data augmentation techniques such as translations, horizontal reflections, or increasing and decreasing brightness was used to pre-train the network, as in [55]. The first part of the network is where the features extraction is done. Then, because the pre-training data was artificially transformed, the network will be able to recognise grasped objects independently of their position, orientation, or grasping pressure (equivalent to brightness in RGB images), exploiting the translation-invariant property [57].

**Figure 11:**
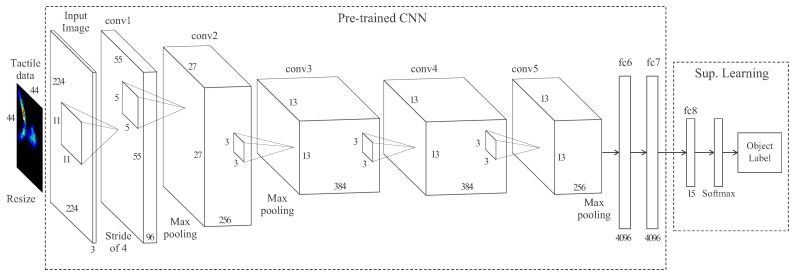
Architecture of the pre-trained deep convolutional neural network (DCNN) with a last custom-trained layer for classification.

The structure of the pre-trained part of the network must be maintained. In our application, the five convolutional layers (conv1-5) and the two first fully-connected layers (fc6 and fc7) comprise the pre-trained part of the network. It is followed by a custom-trained fully-connected layer (fc8) with an output of a 15-way softmax which provides a probability distribution over the 15 classes of objects. Figure 11 illustrates the architecture of the DCNN used for the tactile recognition task.

Because the first convolutional layer of AlexNet filters the 224×224×3 input image, the 44×44×1 input tactile data is resized to 224×244 and copied in each of the three channels. After each convolution, a rectified linear unit (ReLU) introduces non-linearities to the model. A normalization layer with hyper-parameters $\alpha =10^{-4},\;\beta =0.75,\;k=1$, and n=5 is introduced after convolutional layers *conv1* and *conv2*. Then, 3×3 max-pooling layers with a stride 2 follow both the normalization and the fifth convolutional layers. The neurons in the pre-trained fully-connected layers are connected to all neurons in the previous layer, and its outputs are given by ReLUs including a 0.5 probability of dropout. The classifier was implemented in *Matlab R2017b* using the *Neural Network Toolbox* and uploaded to the author’s public software repository (https://github.com/TaISLab/Classification-Tactile-Adaptive-Gripper).

### 4.3. Two-Level Classification

After a set of common objects and human limb parts were used to train the object classifier, a more important piece of information for a rescue robot is to know if the grasped body belongs to a human or not. Although this task could be achieved by knowing the highest rated class form the object classifier, a second-level layer was added to further identify the nature of the manipulated body.

**Figure 12:**
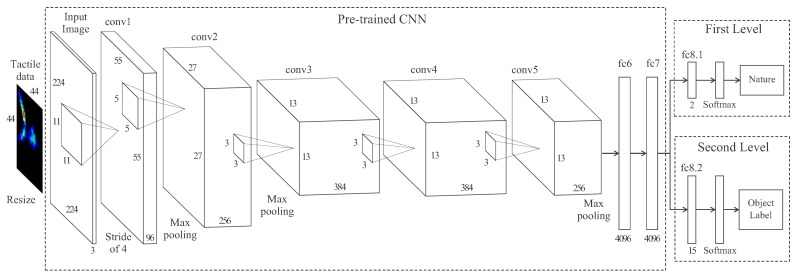
Architecture of the two-level convolutional neural network. The network has two outputs: the first-level classifier (nature) and the second-level classifier (object label).

The architecture of this network is presented in Figure 12. The network has two classification levels. At the first level, a fully-connected layer (fc8.1) with a softmax output provides a probability that the grasped body belongs to a human limb or an inert object, whereas at the second level a fully-connected layer (fc8.2) with a 15-way softmax function returns the probability distribution over the 15 classes of objects. The rest of the architecture of the network and the hyper-parameters match the previous network.

## 5. Experiments

The performance of the different sensor configurations is compared based on the recognition rates of the object classification task. In the experiments, the rigid probe as well as the semi-rigid and flexible grippers were used. To have a comparison point to our proposed method (DCNN), the DCNN-SVM method was also implemented. This method was previously presented in [53], where the first pre-trained layers of a DCNN are used to extract the features of the input tactile images. Then, the SVM replaces the last layer of the network and must be trained with pre-labelled tactile images.

To deeply analyse both the classification method and the gripper configurations, a comparison in terms of mean recognition rate in a 10 and 15 classes experiments with each configuration and with datasets of 20, 30, 40, and 50 tactile images for each configuration are presented. In addition, the training time for each experiment was also evaluated. Furthermore, an evaluation of the recognition rate achieved in the first-level classifier of the two-levels DCNN is done.

The training set was formed by 40% of the images of the dataset for each class—a total of 80, 120, 160, and 200 for the 20, 30, 40, and 50-images dataset respectively for each configuration in the 10 classes experiment; and 120, 140, 240, and 300 for the 20, 30, 40, and 50-images dataset respectively with 15 classes. Note that the experiment’s accuracy depends on the images used for the learning and test phases of the algorithm. Because in this case the number of images was small, the dependency was high and the results could vary depending on the formation of the training and the test sets. For this reason, 20 samples for the dataset of each configuration were evaluated. For each one, the dataset was randomized before being split into the training and test sets. The custom-trained parts of the DCNNs were trained on a GPU NVidia GeForce GTX 1050 Ti with 4 GB of RAM and with learning rate of $10^{-4}$ and momentum of 0.9.

After the training process is done, the classifier result must be evaluated. To do that, the test set—comprised of the remaining 60% of the images of the dataset for each class—is used as an input. Each image of the test set passes through the DCNN, returning a label. This output is then checked with the real label of the input. A confusion matrix for each dataset is obtained, doing this for every image of the test set.

**Figure 13:**
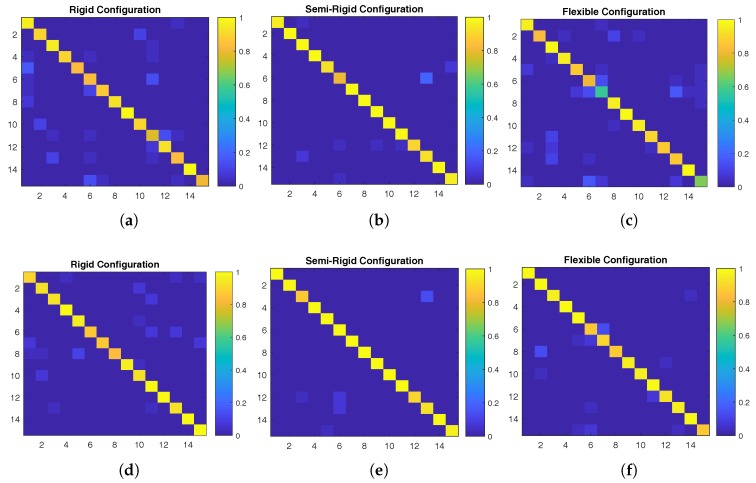
Confusion matrices corresponding to the best recognition rate achieved in the 15-class experiment for the (**a**,**d**) rigid, (**b**,**e**) semi-rigid, and (**c**,**f**) flexible configurations using the DCNN-SVM (support vector machine classifier) (**top**) and DCNN (**bottom**) approaches and the dataset of 50 images per class.

The respective confusion matrices for the samples corresponding to the best recognition rate of each configuration using the DCNN-SVM and the DCNN methods in a 15-class recognition experiment, with a dataset of 50 images per class and per configuration (total of 2250 images) are shown in Figure 13.

**Figure 14:**
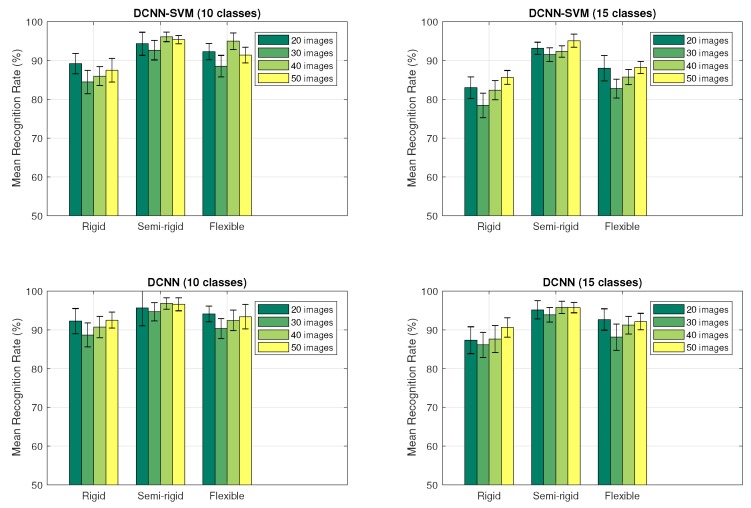
Mean recognition rate of each configuration with (**top**) the DCNN-SVM and (**bottom**) the DCNN approaches for the object recognition experiment with (**left**) 10 and (**right**) 15 classes.

To evaluate the mean recognition rate achieved for each configuration over the 20 samples, a bar graph was obtained from the recognition rate of each sample. This data was then calculated as the average of the diagonals of each confusion matrix. Figure 14 presents the distribution graphs of the accomplished recognition rates by each configuration in the 10 and 15 classes experiments, using the datasets of different sizes corresponding to the application of the DCNN and DCNN-SVM methods to the 20 randomized samples of each dataset.

It can be noticed that—independent of the number of classes of the experiment—the best recognition rate (96.81% in the 10 classes experiment and 95.78% in the 15 classes experiment) was achieved when using the semi-rigid gripper, whereas the worst recognition rate was obtained when using the rigid probe (84.47% in the 10 classes experiment and 78.43% in the 15 classes experiment).

**Figure 15:**
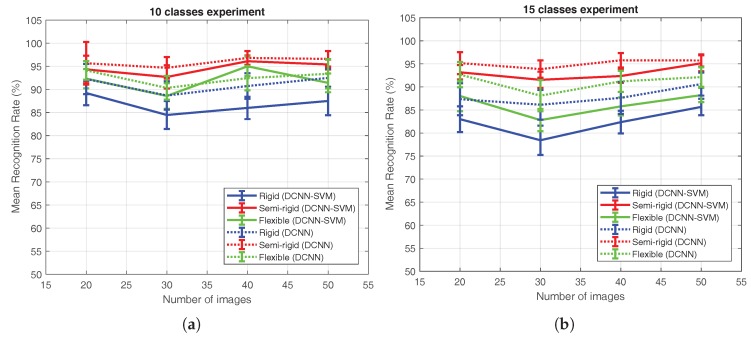
Mean recognition rate and standard deviation of (**a**) the 10 classes and (**b**) 15 classes experiments of the different approaches and configurations with respect to the number of images employed to train the algorithm.

In Figure 15, a deeper comparison of the DCNN and DCNN-SVM methods after the 10 and 15 classes experiments is presented. As commented before, the best accuracy was obtained by the DCNN approach with the semi-rigid configuration, and the worst was obtained by the rigid sensor and the DCNN-SVM method.

**Figure 16:**
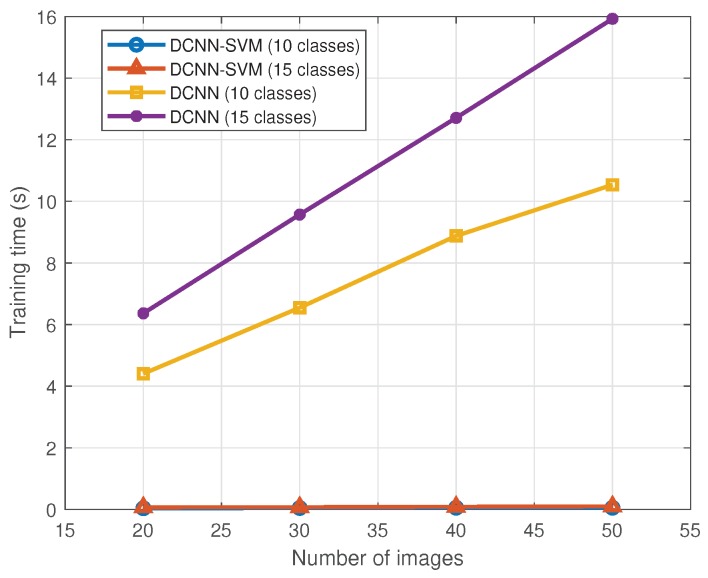
Training time of the different methods and experiments with respect to the number of images.

The DCNN-SVM method needs less time to train than the DCNN. Figure 16 shows the differences in this term. As expected, the highest time was obtained in the 15 classes experiment with the dataset of 50 images per class, using the DCNN method (≈16 s). The lowest time was obtained in the 10 classes experiment with the 20 images dataset per class, using the DCNN-SVM method (≈0.04 s). Note that in order to quantify the training time we have not considered the time needed for the pre-training part of the DCNN.

**Figure 17:**
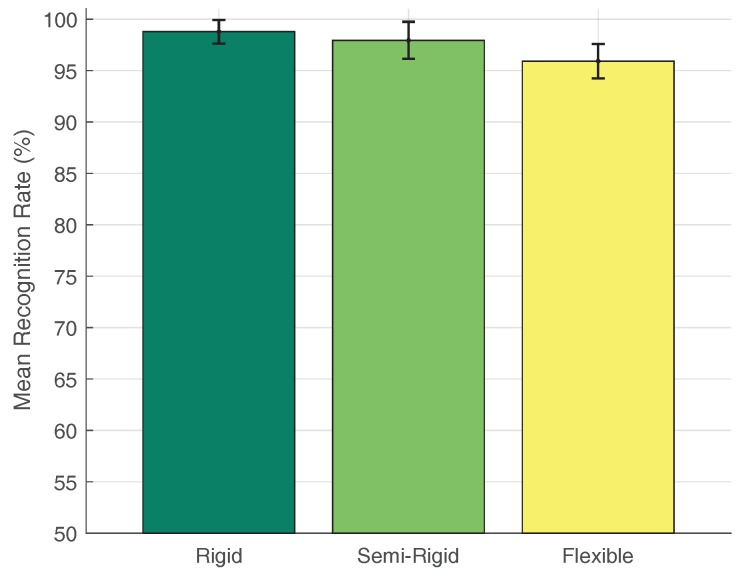
Recognition rate of the first-level output.

Moreover, the two-level DCNN was tested. Note that the second-level output of the network was already studied in the previous experiment (15 classes experiment). On the other hand, the first-level classifier was evaluated using a dataset of 750 tactile images per configuration, where 250 belong to human limbs and the remaining 500 belong to common objects. Figure 17 shows the bar graph resulting from this experiment. In this case, the highest recognition rate (98.78%) was obtained with the rigid configuration, and the worst (95.92%) with the flexible configuration.

## 6. Discussion

The use of tactile sensors in adaptive grippers is an interesting tool, not only to design recognition methods, but also to study the pressure profiles of the robotic grasper. In this case, the pressure profiles of the three configurations were studied showing significant variations in terms of contact area and pressure levels. Although the sensor does not cover the whole contact surface of the gripper (42%), an increment in the contact surface area (26% and 37%), reducing the mean pressure value on the grasped object (57% and 72%) can be seen. This is particularly interesting for the human–robot interaction, as a safe contact requires low-pressure contacts.

The performance of the use of the above mentioned pressure images must be evaluated. Different parameters were studied to analyse the effects of the number of classes, the number of training images, and most importantly the performance comparison between the three gripper configurations. As a result, the best results came from the semi-rigid gripper (up to 96.81%). The figures show that the increment in the number of training images gave lower variance in the results. The increment in the number of classes gave little decrement (2%) of the recognition rate.

When comparing the pure DCNN method with the mixed DCNN-SVM classifier, the results were similar with a consistently higher score in the case of the first (5–6%). The main difference was in the training times, where SVM was much faster (300 times), although this is a one-time operation.

Even when the nature of the classified object could be determined by the object class (see Table 3), a second DCNN was implemented to to have a more generic classification (first level), in the case where the grasped object does not belong to any of the trained classes. This classifier was also evaluated with a high recognition rate (up to 98.78%) for the trained objects. However, the results show that the best sensorial configuration for the detection of human contact is the rigid probe. This can be explained by the better dynamic range in the pressure images, which better distinguishes the texture differences between human skin and objects.

## 7. Conclusions

In this work, the detection capabilities of a robot for pHRI were enhanced thanks to deep learning convolutional neural networks and the use of tactile sensing. In this way, a robot is able to classify the grasped objects based on the pressure images and knowing if it is holding a human body part or an object from the environment. This is particularly useful in poor visibility conditions, which can be common in emergency scenarios, and increases the safety of the human/machine interaction.

To our best knowledge, this work has presented the first adaptive gripper which integrates a flexible tactile sensor for object recognition. Furthermore, the paper has offered results that can be useful for the development of safe manipulation in autonomous robotic systems in contact with humans in catastrophe scenarios. In particular, the pressure profiles from different grippers were measured, showing how a flexible gripper increases the contact area and reduces the pressure on the potential human victims. The accuracy of the tactile detection has also been evaluated in experiments with a specific set of objects and body parts. According to the results, the best configuration for each classification was found.

The results of the experiments have shown that the semi-rigid gripper obtained the best recognition rate when identifying different objects and body parts. However, for the human/non-human classification problem, the best accuracy was obtained by using the rigid gripper. Despite this fact and considering that the difference in the accuracy achieved by these configurations was less than 1%, the authors believe that the use of the semi-rigid gripper is more appropriate during a pHRI due to the benefits of using soft or adaptive grippers both for the safety of the operation and the easier grasping. As mentioned before, the results show how adaptive grippers provide safer contact while keeping high recognition rates using integrated tactile sensors.

Additionally, to ensure the reproducibility of the experiments, all the details about the components used have been included, and the designs, the used datasets, the neural nets, and algorithms presented herein have been made available on-line to the reader.

In future works, the complete integration of the tactile sensing with a manipulation controller for active compliance will be considered, so a safe interaction can be achieved to perform human limbs repositioning and radial pulse measurement.

## Figures and Tables

**Table 1:** Specifications of the tactile sensor model 5051 from *Tekscan*.

Parameter	Value
Max. pressure	48 KPa
Number of tactels	1936
Tactels density	62 tactels/cm${}^{2}$
Temperature range	$-40{\;}^{\circ }$C to $+60{\;}^{\circ }$C
Matrix height	55.9 mm
Matrix width	55.9 mm
Thickness	0.102 mm

**Table 2:** Mechanical properties of the silicon rubber pad used on the contact surface of the gripper fingers.

Parameter	Value
Thickness	3 mm
Density	0.25 g/cm${}^{2}$
Elongation	100%→200%
Temperature range	$-60{\;}^{\circ }$C to $+200{\;}^{\circ }$C

**Table 3:** Fifteen different classes were defined, categorized by their individual object label and nature (Human/Non-human).

Object Label	Nature	Dimensions
Adhesive tube	Non-human	32×45×110 mm
Ball	Non-human	65 mm Ø
Bottle	Non-human	60–85 mm Ø
Box	Non-human	22×22 mm section
Branch	Non-human	18–22 mm Ø
Cable	Non-human	n.a.
Can	Non-human	66 mm Ø
Mouse	Non-human	62×108×48 mm
Rock	Non-human	37×70×98 mm
Screwdriver	Non-human	19–31 mm Ø
Tape	Non-human	53 mm Ø
Arm	Human	50–70 mm Ø
Fingers	Human	16×70 mm
Hand	Human	30×80 mm section
